# Examining the use of Amazon’s Mechanical Turk for edge extraction of the occlusal surface of fossilized bovid teeth

**DOI:** 10.1371/journal.pone.0179757

**Published:** 2017-07-13

**Authors:** Gregory J. Matthews, George K. Thiruvathukal, Maxwell P. Luetkemeier, Juliet K. Brophy

**Affiliations:** 1 Department of Mathematics and Statistics, Loyola University Chicago, Chicago, IL, United States of America; 2 Department of Computer Science, Loyola University Chicago, Chicago, IL, United States of America; 3 Department of Geography and Anthropology, Louisiana State University, Baton Rouge, LA, United States of America; Institut Català de Paleoecologia Humana i Evolució Social (IPHES), SPAIN

## Abstract

In order to reconstruct environments associated with Plio-Pleistocene hominins in southern Africa, researchers frequently rely upon the animals associated with the hominins, in particular, animals in the Family Bovidae. Bovids in southern Africa are typically identified by their teeth. However, identifying the taxon of a bovid tooth is challenging due to various biasing factors. Furthermore, inaccurate identification of fossil bovids can have significant consequences on the reconstructed paleoenvironment. Recent research on the classification of bovid fossil teeth has relied on using elliptical Fourier analysis to summarize the shape of the outline of the occlusal surface of the tooth and the resulting harmonic amplitudes. Currently, an expert in the field must manually place landmarks around the edges of each tooth which is slow and time consuming. This study tests whether it is possible to crowdsource this task, while maintaining the necessary level of quality needed to perform a statistical analysis on each tooth. Amazon Mechanical Turk workers place landmarks on the edge of the tooth which is compared to the performance of an expert in the field. The results suggest that crowdsourcing the digitization process is reliable and replicable. With the technical aspects of digitization managed, researchers can concentrate on analyzing and interpreting the data.

## 1 Introduction

Reconstructing past environments associated with early hominins is essential for understanding human evolution and is valuable for identifying habitat preferences, diet, and ecological relationships between hominins and other species. In order to reconstruct past environments, paleoanthropologists commonly rely on the animals that are found associated with the hominins. Animals in the Family Bovidae such as antelopes and buffalo are particularly useful for this task due to their strict ecological tendencies [1–3]. In addition, bovids are one of the most common fossils found in southern Africa, in particular isolated teeth. However, identifying bovid teeth in the fossil record is complicated by biasing factors such as attrition and sex [4]. Overlap exists in the form (i.e. size and shape) of bovid teeth making it difficult to identify the taxon and, therefore, difficult to reconstruct the past environment [4]. The purpose of this study is to demonstrate a reliable, replicable, uncomplicated method for extracting the form of the occlusal surface of bovid teeth which can then be used to identify teeth in the fossil record. Several recent studies have demonstrated that morphometrics is particularly useful for documenting biological shape [5–11]. This new methodology extracts edges by relying on crowdsourcing. The outlines are then used in supervised machine learning techniques in conjunction with elliptical fourier analysis (EFA) [12].

It should be noted that ideally edge extraction of the occlusal surface of these teeth could be performed using automated procedures based on techniques such as those described in [13] or [14]. However, in this specific setting automated methods are difficult to use as these techniques tend to often identify the bottom of a tooth as the edge rather than the actual occlusal surface.

Previously, [1] performed a study to standardize the identification of bovid teeth using EFA. While successful in identifying bovid taxa, the process to extract the outlines was tedious and time consuming. In order to extract the outline of a tooth, an image was imported into a digitizer program, MLmetrics [15], where 60 points were manually placed around the tooth according to a template so as to maintain homology. The points were then exported and analyzed in a fourier analysis program [16]. The study generated occlusal outline information for over 7000 extant and fossil teeth. However, the results could not be easily used to identify fossils from new sites due to the time consuming nature of the process of edge extraction. The present study provides results of an exploratory analysis that employs Amazon’s Mechanical Turk platform [17] as a method to crowdsource the edge extraction of bovid teeth.

In this study, the digitized outlines of an expert in the field, the co-author Juliet K Brophy (JKB), are compared with up to three outlines extracted by Amazon Mechanical Turk workers. The results of this preliminary study suggest that crowdsourcing the digitizing process is reliable and replicable. Furthermore, this streamlined process allows for more teeth to be processed in a timely manner, saves the time of researchers from performing technical tasks, and frees them up to focus more of their time on aspects of this project that require expertise, such as analyzing and interpreting the data.

## 2 Related work

Mechanical Turk [17] was introduced by Amazon.com, Inc. in 2005. As such, there is a relatively limited body of scholarly work exploring the uses of the platform. The projects that task quality assessment, the focus of this study, can be divided into two categories: assessing survey response accuracy and annotating digital images.

### 2.1 Assessing survey response accuracy

Studies in this category focus on investigating how accurate survey responses are from Mechanical Turk Workers. These analyses aim to answer questions such as: How closely do Mechanical Turk surveys reflect surveys distributed using more traditional methods? [18]; How honest are Mechanical Turk workers in their responses? [19, 20]; and Does Mechanical Turk provide researchers with a more diverse response pool than the mainstay of distributing surveys to college students with the promise of extra credit? [21]

[20] uses Mechanical Turk in order to combine the speed and cost-effectiveness of a simulated study with the authenticity of human behavioral studies when analyzing human cooperation. The study claims that prior to Mechanical Turk and the ability to crowdsource data collection, most evolutionary models were based on simulations or mathematical algorithms due to the lack of survey labs and a consistent subject pool. With its use, however, researchers can request a task to be done and collect results entirely online much in the same way a simulation study is conducted. With that said, [20] mentions that a major concern of using Mechanical Turk is the lack of control researchers have over their subjects. It is possible, for instance, for subjects to incorrectly answer a question due to a lack of understanding. Additionally, subjects are completely free to leave in the middle of the survey. After conducting a number of experiments, both online and in person, [20] found that these limitations had a very small effect on the results.

In a similar study, [21] conducted an experiment comparing the performance of Mechanical Turk workers versus subjects in a controlled laboratory setting in an acceptability judgment task. The main concern addressed in [21] is that additional noise, introduced by using Mechanical Turk, might detract from the power of the experiment. To help control for this, they introduced a rejection criteria. Mechanical Turk workers were required to be native English speakers, which resulted in a 15% rejection rate. [21], like [20], states that another major concern in the use of Mechanical Turk is the inability to establish whether or not the Turker understood the task, possibly resulting in inaccurate data. It concluded, however, that using Mechanical Turk is comparable to laboratory research as long as a mechanism exists to reject certain responses.

Additional information on testing best practices when using Mechanical Turk in survey research can be found in [19], which evaluates how various factors effect the reliability of responses, and [18], which compares the demographics of Mechanical Turk respondents to national demographics.

### 2.2 Annotating digital images

This category of Mechanical Turk work evaluates the quality of edge extraction research. Two of the primary works related to this topic include [22] and [23].

[22] explored the use of Mechanical Turk in image classification focusing on techniques for automatically “cleaning” the data sets. They demonstrate that by using multiple methods for measuring the accuracy of annotations they can outperform other methods that rely on a single measure. They also demonstrate that image classification can be performed with high levels of accuracy when using Mechanical Turk workers to extract the edge of images. Further, classification accuracy can be improved by over 7%, by cleaning the data using the techniques considered in this study.

[23] evaluates various annotation techniques with the goal of maximizing quality while minimizing cost. This research used landmark-based edge extraction and a gold standard method of grading. Landmark extraction, or annotation, involves having a Turker place a number of points along the border of an image. Once the outline is extracted, it can be tested for quality against an outline annotated by an expert, which is referred to as the “gold standard” grading technique. While it was not used in this particular study, [23] also mentions grading outlines based on their distance from the mean image produced by multiple Mechanical Turk workers, which may be useful as it eliminates the need for expert tracing.

## 3 Methods

This exploratory study includes a sample of 96 teeth of known species from four different tribes: Alcelaphini, Bovini, Hippotragini, and Neotragini. These teeth were obtained from the Ditsong Museum (TM) (formerly Transvaal Museum) and the National Museum of Bloemfontein (NMB), South Africa. (Permission to use these specimens was received by JKB from both institutions (i.e. National Museum, Bloemfontein and Ditsong Museum (formerly Transvaal Museum)). Permits are not required to look at extant bovid specimens in South Africa. Therefore, no permits were required for the described study.) The complete repository information is in Table 1. Permission was received from each institute to photograph these specimens. No permits were required for the described study, which complied with all relevant regulations.

**Table 1:** List of extant bovid specimens used in this study from the National Museum, Bloemfontein (NMB) and the Ditsong Musuem (TM) (formerly Transvaal Museum).

Genus	Species	Repository	Specimen Number
**UM3**			
*Connochaetes*	*taurinus*	NMB	64, 12066, 12204, 12475
*Damaliscus*	*dorcas*	NMB	8752, 9382, 12159, 12175, M144
*Oryx*	*gazella*	NMB	250, 9304, 9330, 12094, 12181
*Hippotragus*	*niger*	NMB	177, 178, 183, 232, 893
*Syncerus*	*caffer*	NMB	9, 12, 16
**UM2**			
*Hippotragus*	*equinus*	NMB	191, 196, 887
*Hippotragus*	*equinus*	TM	AZ 1133
**LM3**			
*Alcelaphus*	*buselaphus*	NMB	6022, 8715, 8763, 12199, 12215
*Raphicerus*	*campestris*	NMB	8730, 9343, 9438, 9761, 9787
*Pelea*	*capreolus*	NMB	9446, 6878
*Pelea*	*capreolus*	TM	AZ 479, 10005, 10007
*Syncerus*	*caffer*	NMB	1000, 1001, 1002, 8743, 8774
**LM2**			
*Connochaetes*	*gnou*	NMB	12218, 12323, 12399, 12394
*Connochaetes*	*taurinus*	NMB	12201, 12204, 12209, 12475, 12476
*Alcelaphus*	*buselaphus*	NMB	6022, 12196, 12199, 12215, 12420
*Damaliscus*	*dorcas*	NMB	7440, 9384, 12039, 12157
*Raphicerus*	*campestris*	NMB	8730, 9438, 9761, 9787, 12169
*Pelea*	*capreolus*	NMB	6878, 9446, 9855
*Oryx*	*gazella*	NMB	9304, 9335, 12182, 12213, 12352
*Hippotragus*	*niger*	NMB	176
*Hippotragus*	*niger*	TM	3812, 4251, 13130, 13136
*Hippotragus*	*equinus*	NMB	191, 887
*Hippotragus*	*equinus*	TM	AZ 2444, AZ 1333, 12072
**LM1**			
*Alcelaphus*	*buselaphus*	NMB	8790, 12195, 12420
*Damaliscus*	*dorcas*	NMB	9384, 12039, 12157, 12320, 15155
*Hippotragus*	*niger*	TM	3812, 13130, 13138, 13143, 13153

**Fig 1:**
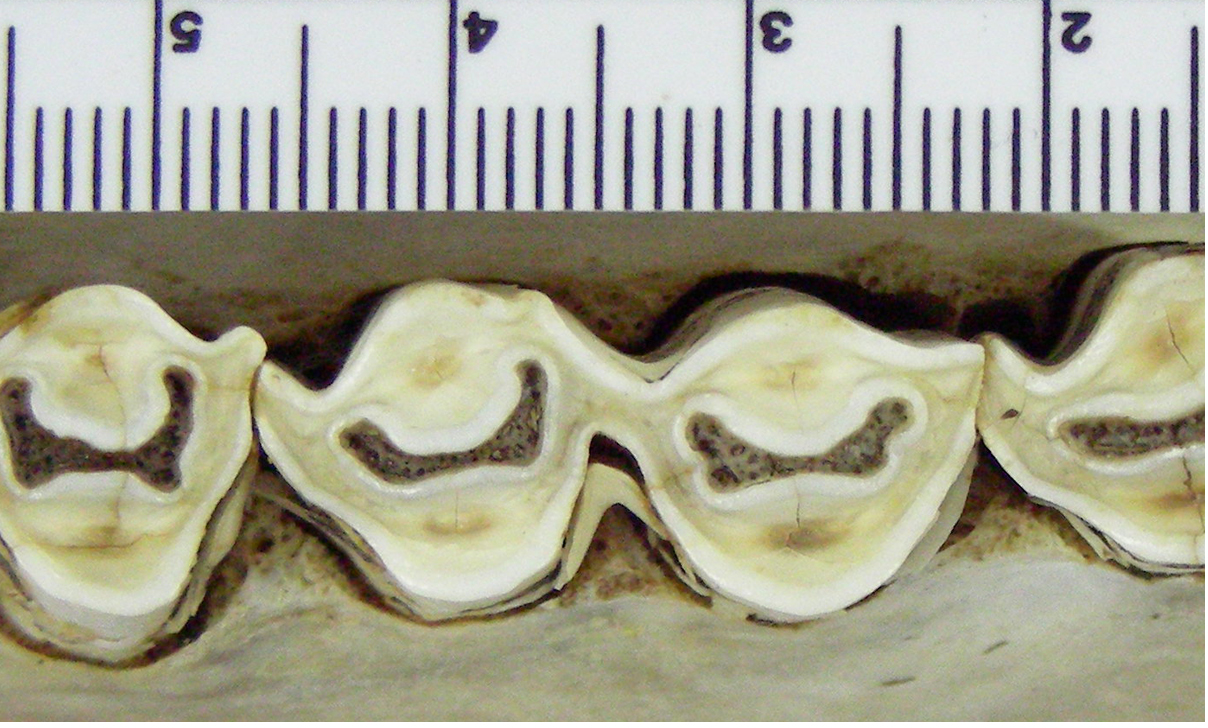
Raw image of tooth.

We investigated three mandibular molars (LM1, LM2, LM3) and two maxillary molars (UM2, UM3). Details of the data are shown in Table 2. An example of the raw image of a tooth prior to extraction can be seen in the left side of Fig 1. Prior to being digitized by a Turk worker, all of the teeth were scaled to each other.

**Table 2:** The distribution of tribe by tooth type in the data set.

	Tooth Type					
Tribe	LM1	LM2	LM3	UM2	UM3	Total
Alcelaphini	8	15	5	0	5	33
Bovini	0	0	5	0	5	10
Hippotragini	5	15	0	5	10	35
Neogtragini	0	8	10	0	0	18
Total	13	38	20	5	20	96

### 3.1 HIT protocol

Amazon states: “A Human Intelligence Task, or HIT, is a question that needs an answer. A HIT represents a single, self-contained task that a Worker can work on, submit an answer, and collect a reward for completing” [24]. Specifically in this setting, the Mechanical Turk worker downloads the image of a bovid tooth in the freeware GIMP (the GNU Image Manipulation Program) [25]. After testing several programs for obtaining the polygon, this program produced the best results. Next, the Mechanical Turk worker selects the *lasso tool* which allows a polygonal selection to be made around the tooth. Once the bounding polygon has been created, the user then cuts and pastes the extracted selection onto a blank canvas. This shape is then filled in with all black using the bucket fill tool in GIMP creating a black and white image of each tooth where the interior of the tooth is black and the background is all white. The resulting file is then saved onto one’s computer and uploaded to the link provided in the HIT.

### 3.2 Processing the Mechanical Turk output

For every raw image of a tooth considered in this study, Mechanical Turk workers were asked to extract the outline of the occlusal surface in GIMP [25]. This process was repeated 3 times for each tooth. (Mechanical Turk workers were used only to trace images of bovid teeth. No personal information relating to any mechanical Turk worker was collected.) The output from each of the Mechanical Turk workers was then forced to a black and white image using ImageMagick [26]. The expert (JKB) also traced the outline of the occlusal surface of each tooth using the exact methods as the Mechanical Turk workers.

**Fig 2:**
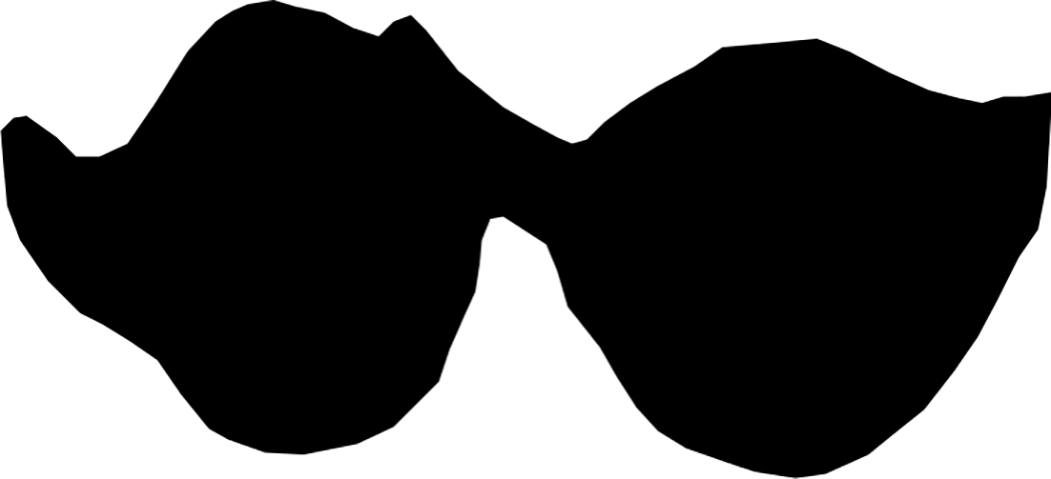
Extracted Occlusal Surface using GIMP performed by expert.

**Fig 3:**
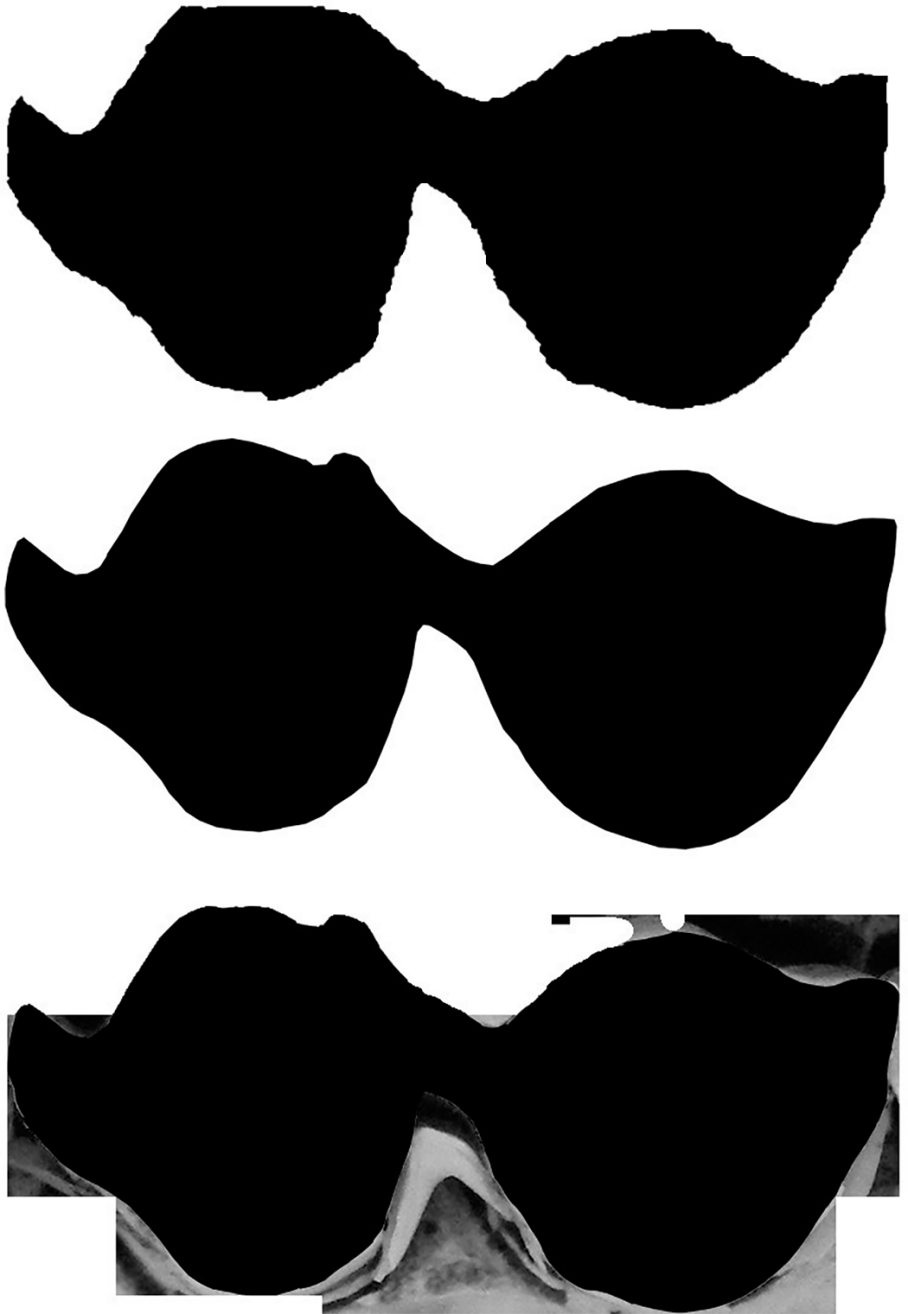
Three black and white images produced by Mechanical Turk workers for the tooth shown in Fig 1.

Fig 1 shows an example of a raw image of a tooth, and Fig 2 shows the tracing by an expert using GIMP. The results from Mechanical Turk workers for this specific tooth are shown in Fig 3. Each of these three images corresponds to different tracings of the raw tooth presented in Fig 1. Note that the tracing on the bottom of Fig 3 was not done correctly by the Mechanical Turk worker and needed to be adjusted after the fact to an image that is strictly black and white.

While three separate HITs for each tooth were posted, we were not always able to get three viable tracings. In some cases, no tracing was returned whereas is other cases, tracings were returned but were clearly wrong. This circumstance occurred, for instance, when Turkers traced around the occlusal surface of teeth that were not the focus of the image, traced some parts of the internal area of the tooth, or returned a.xcf file (a GIMP file) instead of the .jpg that was requested in the HIT.

Once the black and white images are collected and processed, they can be read into R using the “import_jpg” function from the Momocs package [27]. This function extracts x- and y- coordinates along the border between the black and white fields in the images returned from Mechanical Turk workers.

A restriction of the “import_jpg” function is in how an image’s points are sequenced the same from tooth to tooth. So, for instance, the first point listed for one tooth could correspond to the extreme left of the image and the first point listed for a different tooth could be the point on the extreme right. This creates problems when using landmark based approaches such as calculating distances between Mechanical Turk workers and the gold standard teeth extracted by an expert. In order to overcome this, we took the points extracted from “import_jpg” and first performed EFA using the function “efourier” on the (x, y)-coordinates to derive harmonics that describe the tooth.

Elliptical Fourier Analysis is specified as a parametric function

$$\begin{array}{c} x=f\left(t\right)=A_{0}+\sum_{j=1}^{H}a_{j}cos\left(jt\right)+\sum_{j=1}^{H}b_{j}sin\left(jt\right) \end{array}$$

$$\begin{array}{c} y=f\left(t\right)=C_{0}+\sum_{j=1}^{H}c_{j}cos\left(jt\right)+\sum_{j=1}^{H}d_{j}sin\left(jt\right) \end{array}$$

where *H* is the number of harmonics used, *A*_0_ and *C*_0_ are constants, and *a*_*h*_, *b*_*h*_, *c*_*h*_, and *d*_*h*_ are the amplitudes associated with the *h*-th harmonic and *h* = 1, 2, ⋯, *H*. Since EFA is not a landmark based procedure, the initial ordering of the points does not hinder the estimation of the harmonics. Next, so that we are able to perform landmark based analysis, we used the estimated harmonics to output a specific number of points around the edges of each tooth which all begin in the same location. These resulting points act as landmarks, which were used to calculate Riemann distance between shapes created by Mechanical Turk workers and created by the expert.

Additionally, the amplitudes (i.e. *a*_*h*_, *b*_*h*_, *c*_*h*_, and *d*_*h*_) created in EFA can then be used as input features in machine learning algorithms to classify the teeth to tribes and species. Since ultimately what we are interested in is classifying these teeth, the performance of classifiers based on the work of Mechanical Turk workers was compared to the classification accuracy when the model was trained using the outlines traced by the expert. The classification algorithm considered here was random forests [28]. The tracings from the Mechanical Turk worker and the expert were compared to assess how similar they are and to asses differences in the predictive accuracy.

In order to measure the tracing error, Riemanian distance [21] was calculated between the Turkers tracings and the expert tracing. To do this, we first extracted the edges of the black and white images using the “import_jpg” function in the “Momocs” [19] package in R. This creates a given number of (x, y)-coordiates for the outlines of the black and white images. However, the ordering of these points may not line up correctly with the ordering of another tracing of the same tooth. These harmonics can then be used as input in the function “efourier_shape” to output 150 (x, y)-coordinates which act as landmarks around each tooth so that a direct comparison can be made between the mechanical Turk tracings and the tracings performed by the expert.

### 3.3 Evaluation of Mechanical Turk work

In order to measure the tracing error, Riemanian distance [29] was calculated between the tracings generated by Turkers and the expert tracing. To do this, we first extracted the edges of the black and white images using the “import_jpg” function in the “Momocs” [27] package in R [30]. This creates a given number of (x, y)-coordiates for the outlines of the black and white images. However, the ordering of these points may not line up correctly with the ordering of another tracing of the same tooth. These harmonics can then be used as input in the function “efourier_shape” to output 150 (x, y)-coordinates which act as landmarks around each tooth so that a direct comparison can be made between the mechanical Turk tracings and the tracings performed by the expert.

Ultimately the goal of tracing these outlines is to accurately classify the tribe and species that these teeth represent. Previous work [31] compared five different machine learning algorithms based on their performance classifying teeth into tribe and species. Here, we only consider the use of random forests for classification of tribe to compare the tracings created by mechanical Turk workers to the tracings created by JKB.

## 4 Results

### 4.1 Tracing error

**Fig 4:**
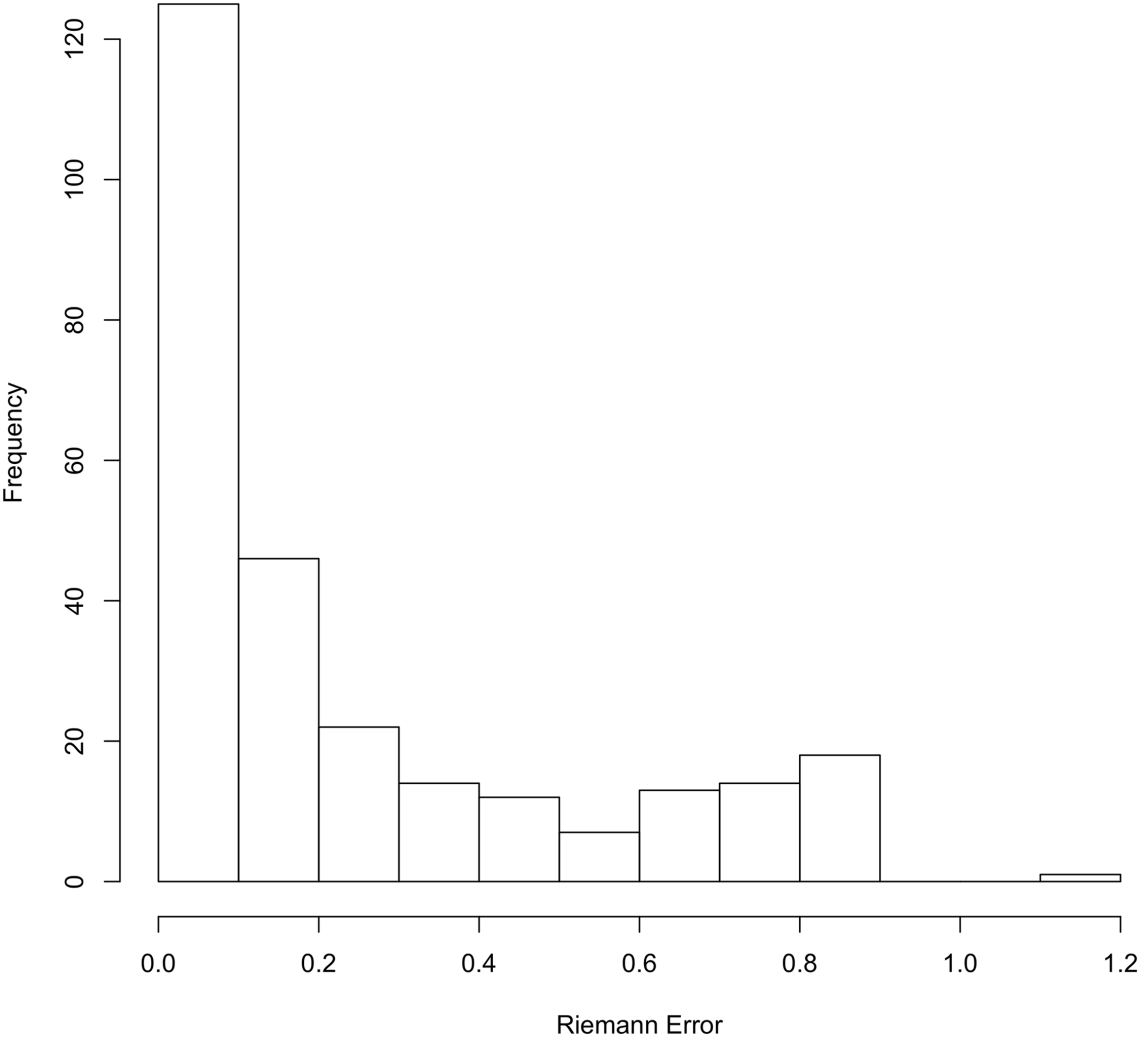
Distribution of errors as measured by Riemannian distance.

**Fig 5:**
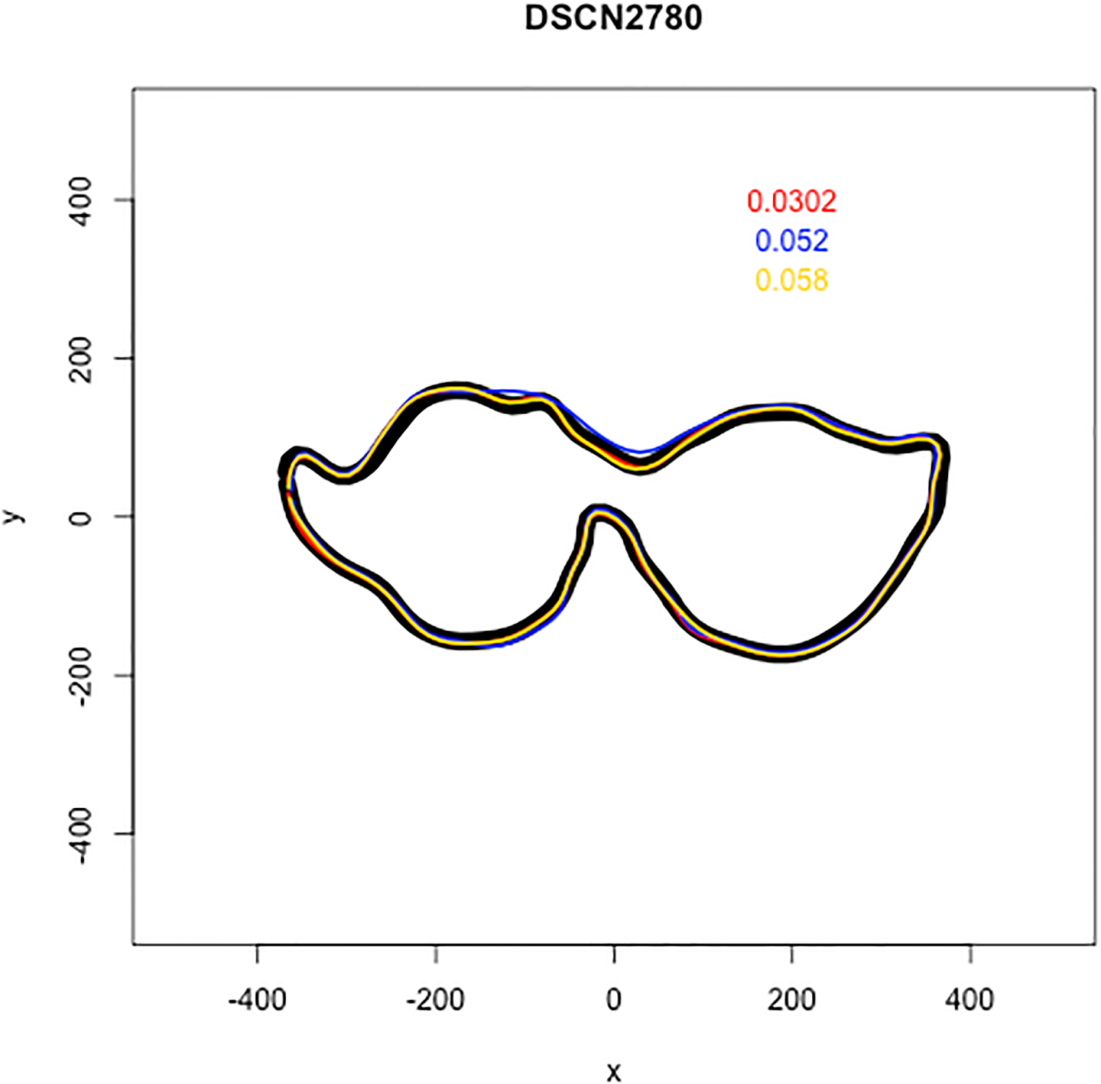
The black outline is the tracing done by JKB and the three other teeth in red, blue, and yellow correspond to the three tracings done by the Mechanical Turk workers. The red, blue, and yellow numbers that appear the upper right of the image correspond to the Riemanian distance between each Mechanical Turk tracing and the tracing done by the expert.

**Fig 6:**
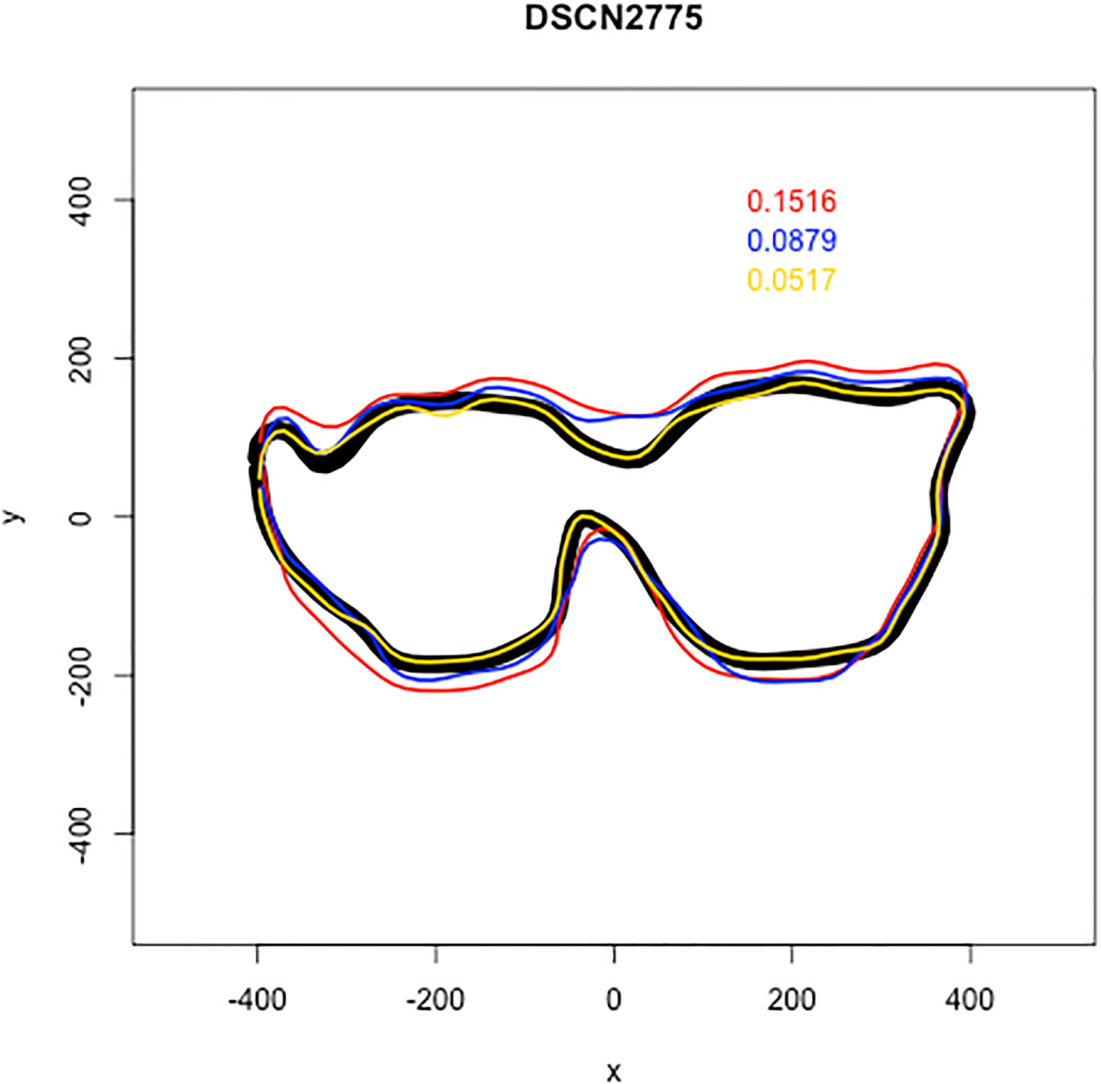
The black outline is the tracing done by JKB and the three other teeth in red, blue, and yellow correspond to the three tracings done by the Mechanical Turk workers. The red, blue, and yellow numbers that appear the upper right of the image correspond to the Riemanian distance between each Mechanical Turk tracing and the tracing done by the expert.

The Riemanian error distances ranged from 0.01113 to 1.113 with a median error of 0.1154. A histogram of this distribution can be seen in Fig 4. Notice that the distribution is skewed heavily to the right and indicates that many of the Mechanical Turk workers trace the outline with only small amounts of error with a full 50% less than 0.1154. For reference, Figs 5 and 6 show two examples of the work of Mechanical Turk workers, with outlines in red, yellow, and blue, compared to the gold standard, which is shown in black. In Fig 5, an example of the results for a tooth is shown. The Mechanical Turk tracings are visually nearly identical to the gold standard tracing and these correspond to Riemann distances of 0.0191, 0.0527, and 0.0342 for red, blue, and yellow, respectively. The other image in Fig 6 displays a different tooth where the Mechanical Turk workers struggled a bit more to accurately trace the outline of the occlusal surface relative to the gold standard. Visually the yellow tracing is the most accurate relative to the gold standard and has the lowest Riemann error of 0.0517. The tracings displayed by the red and blue curves are less accurate and correspond to Riemann errors of 0.1516 and 0.0879, respectively.

**Fig 7:**
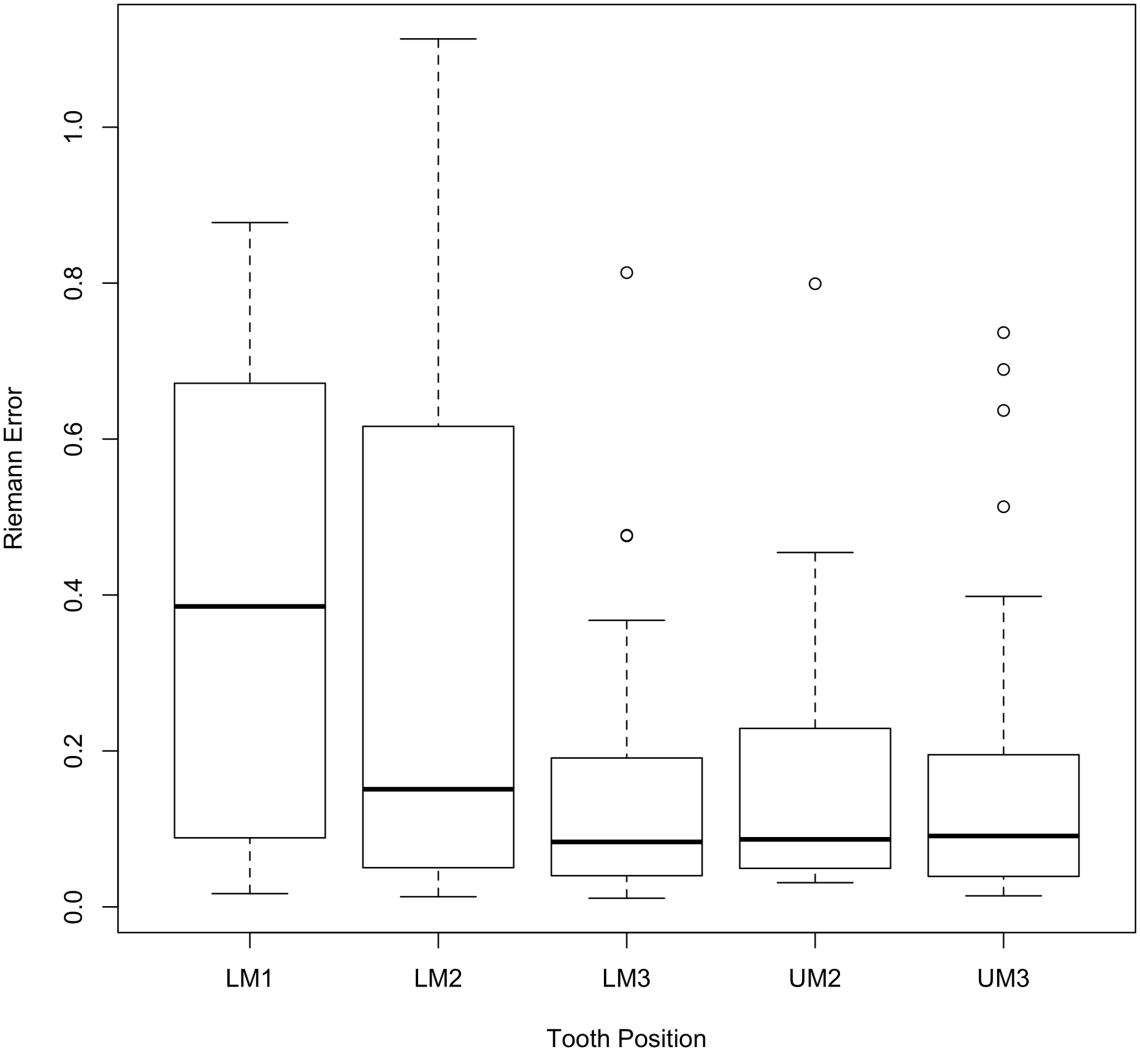
Errors as measured by Riemannian distance by tooth position.

**Fig 8:**
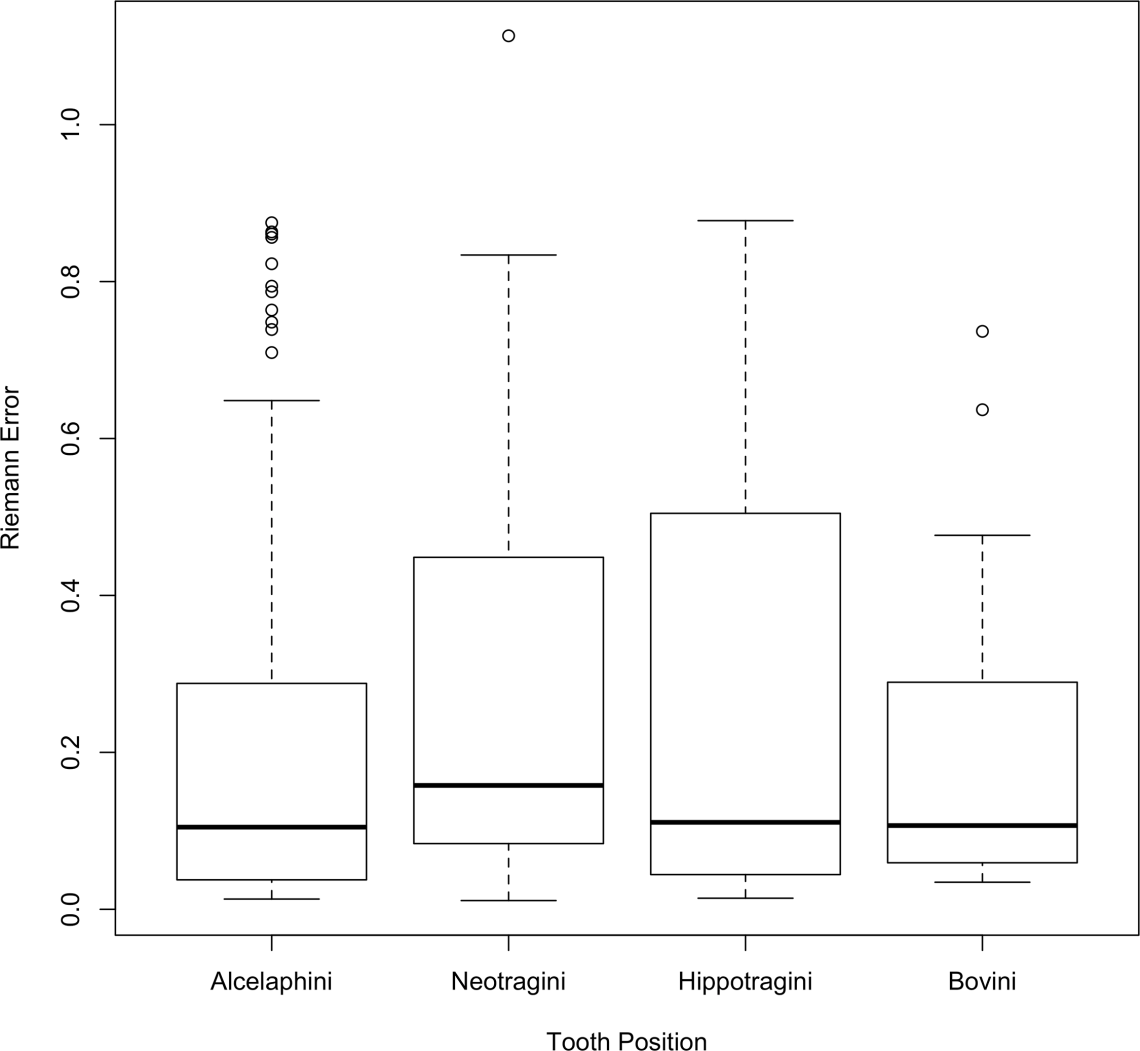
Errors as measured by Riemannian distance by Tribe.

With a frame of reference for the meaning of the Riemann errors, Figs 7 and 8 display boxplots corresponding to the distribution of the Riemann distance by tooth position and Tribe. In Fig 7 it is evident that first lower molars (LM1) have much larger errors between the Mechanical Turk workers and the expert among tooth positions considered here.

### 4.2 Predictive accuracy

**Fig 9:**
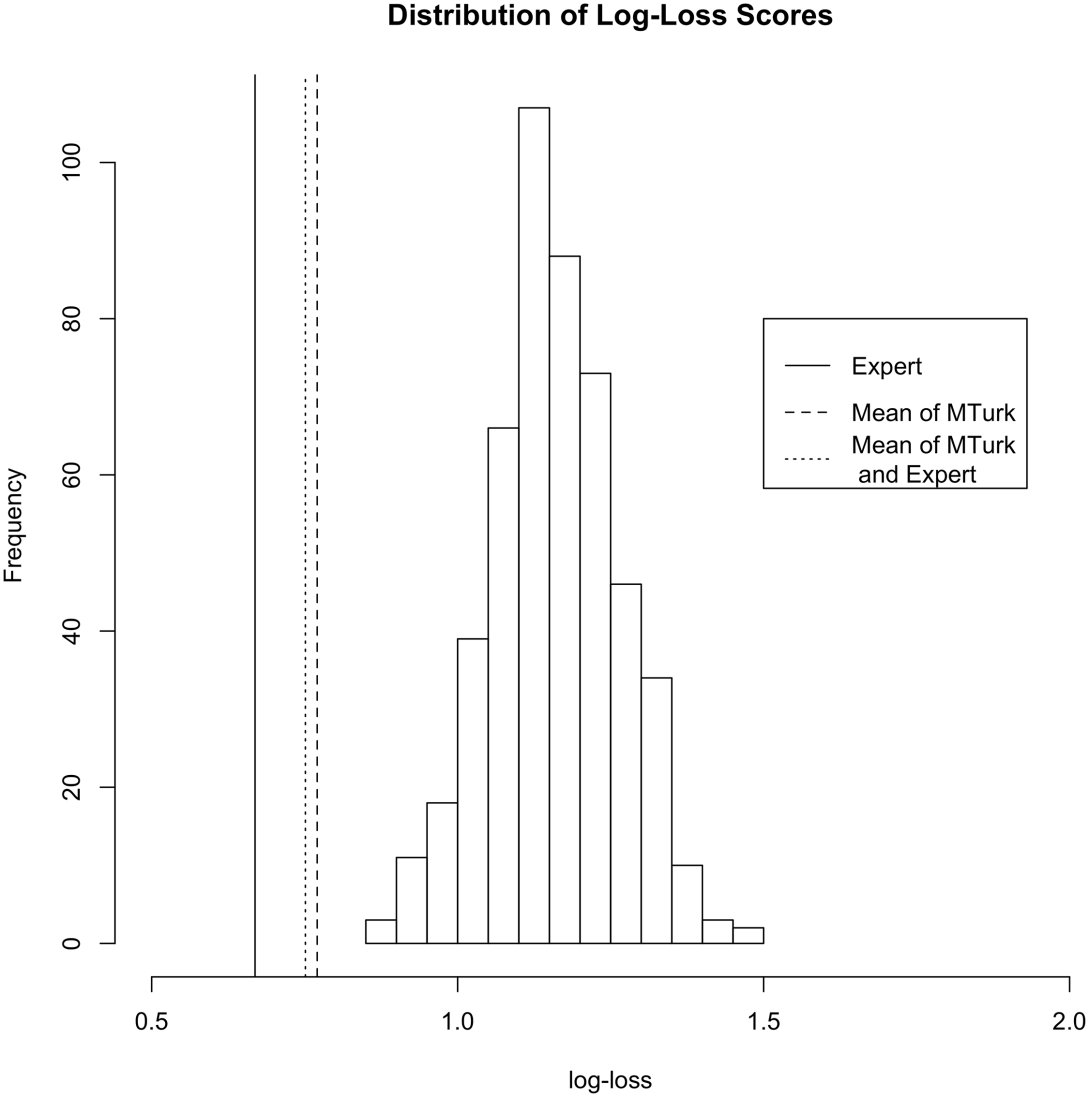
Comparing the classification accuracy of different methods of extracting the edges of bovid teeth.

The histogram seen in Fig 9 depicts the classification accuracy results from the crowdsourced tracings. These results were created by repeatedly sampling one of the at-most three tracings per tooth in order to make a data set. Leave-one-out-cross validation was then performed using random forests. Accuracy of the model was quantified using a log loss score, comparing the predicted class to the actual observed class. From the histogram, it can be seen that if only one Turker for each image was used, they would perform consistently and considerably worse than the expert. The best sample is roughly .85 in terms of log-loss, while the mean is closer to 1.3, while the worst case is nearly 1.5.

The dotted line labeled “Mean of MTurk” was calculated by classifying the average shape of the Turkers outlines after eliminating obviously incorrect tracings. One can see that there is an improvement over even the best sample of individual workers. By taking the average image, the log-loss value lowered to 0.7788 for classifying the tribe.

Using the expert’s tracings we can further reduce log-loss, which is to be expected, down to 0.6689. While this is certainly an improvement over the Mechanical Turk workers, we argue that this level of log-loss is still acceptably close to the expert to still be of use in that the time that is saved by crowdsourcing the extraction of the edges is worth a small trade-off in classification accuracy.

Finally, we evaluated the classification performance of the traced outlines by averaging all of the Mechanical Turk workers (excluding images where the Riemann distance was greated that 0.2 from the expert) and the expert. This slightly improved classification accuracy compared to the average of the Mechanical Turk workers to a log-loss of 0.7524; however, the expert alone still has the lowest log-loss.

Finally, we consider results in terms of misclassification rather than log-loss. Table 3 shows the misclassifications for JKB alone. Using only those tracings, the model was able to classify correctly 79% of the specimens in cross validation. A large amount of the error occurred between Alcelaphini and Hippotragini. Namely, of the missclassified observations, 75% were either actually Alcelaphini but classified as Hippotragini, or actually Hippotragini but classified as Alcelaphini. Table 4 shows the missclassification results of the average image from the Turkers. The model correctly classified the Turker results 74% of the time. Once again, the largest source of confusion was between Hippotragini and Alcelaphini. Table 5 shows the results when the outlines of the Mechanical Turk workers were averaged with the gold standard. Somewhat surprisingly, this result was worse in terms of missclassification than the other two specifications considered here with a classification rate of 68% in spite of being better than using the Turk outlines only in terms of log-loss.

**Table 3:** Expert tracings only.

	Predicted Class		
Actual Class	Alcelaphini	Hippotragini	Neotragini
Alcelaphini	11	3	1
Hippotragini	3	11	1
Neotragini	0	0	8
Log Loss: 0.6688741			

**Table 4:** Mean of MTurk worker tracings (Removed if Riemann distance from mean > 0.2).

	Predicted Class		
Actual Class	Alcelaphini	Hippotragini	Neotragini
Alcelaphini	10	2	3
Hippotragini	4	11	0
Neotragini	1	0	7
Log Loss: 0.7787713			

**Table 5:** Mean of MTurk workers and expert tracings.

	Predicted Class		
Actual Class	Alcelaphini	Hippotragini	Neotragini
Alcelaphini	11	3	1
Hippotragini	5	8	2
Neotragini	1	0	7
Log Loss: 0.7524494			

## 5 Discussion

The results of this study suggest that the proposed method will dramatically decrease the amount of subjectivity in bovid tooth identification and will advance the field of paleoanthropology/zooarchaeology. The importance of this method cannot be understated. As mentioned previously, bovids have different ecological requirements. Therefore, misidentified bovids can lead to incorrect paleoenvironmental reconstructions. For example, three researchers analyzed the bovid fauna from the South African site of Makapansgat and proposed paleoenvironmental reconstructions for Member 3 [32–34]. While each researcher relied upon the same assemblage to form their reconstruction, the papers suggest a different paleoenvironment: shrub-like with nearby open grasslands [32]; woodland [33]; and bushland with riparian woodland and nearby limited wetlands [34]. Reconstructions like these are used to discuss hominin behavior as well as speciation and extinction events. In fact, until recently it was commonly thought that one early human ancestor, Australopithecus robustus, went extinct due to being a habitat specialist that could not survive in fluctuating environmental conditions [4]. By more accurately identifying the bovids from sites associated with A. robustus using morphometrics, [4] was able to demonstrate that this hominin lived in a variety of habitats that changed over time; A. robustus was more likely a habitat generalist. Therefore, the hypothesis that A. robustus went extinct because it was a habitat specialist requires rethinking. If a fraction of these subjectivity problems are solved with this new methodology, the field is advancing and more accurate paleoenvironmental reconstructions and interpretations will be made.

With that said, some preliminary issues exist with this methodology. First, if a large number of teeth needs to be traced with replicates of each tooth, this process can get expensive. In the future, ideally, we will be able to leverage modern computer vision algorithms to extract the edges of these teeth with little or possibly no human aid. Second, some teeth are more difficult for a lay person to trace (e.g. LM1) and those teeth may still require an expert to trace those teeth or at least someone who has received more training than the average Mechanical Turk worker. This result is not unexpected as this method is not designed to completely replace all other forms of tooth identification, rather it is intended to provide objective, reliable classifications of bovid teeth and to supplement and be supplemented by other forms of tooth identification, as needed. Regardless of these problems, the benefits of employing this method and decreasing the subjectively involved in bovid tooth identification far outweigh the issues.

## 6 Conclusion

This study demonstrates that by taking the average shape of multiple Mechanical Turk workers, we can quickly obtain the outline the occlusal surface of a tooth that performs similarly to the expert’s in terms of classification. A database was created of 96 different teeth along with the associated ground truth tracings done by an expert. Once outlines traced by non-experts through Amazon’s Mechanical Turk were collected, we imported an outline into R and lined up landmarks for comparison using EFA. The accuracy of the tracings was evaluated by calculating the Riemann distances between the landmarks on the crowdsourced outline and the outlines generated by the expert. Further, predictive accuracy was assessed using leave-one-out cross validation with random forests on a small subset of the data. We find that in terms of log-loss the tracings performed by the expert, while superior, were not substantially better than using the average of the mechanical Turk workers. In terms of classification accuracy, we measured 74% classification rate using the average of the tracings of the mechanical Turk workers, which is very close to the classification accuracy of 79% when using the tracings generated by the expert. The results suggest that this process can be useful for researchers in many scientific areas (e.g. anthropologists, paleontologists, zooarchaeologists, etc.) who need quick, objective classifications for teeth recovered in the field. Further, one area of future work we are particularly interested in is the analysis and classification of partially observed teeth due (i.e. broken teeth). We believe that this method explored here can be easily extended to the case when teeth are broken.
